# Recovering the superficial microvascular pattern via diffuse reflection imaging: phantom validation

**DOI:** 10.1186/s12938-015-0081-x

**Published:** 2015-09-30

**Authors:** Chen Chen, Klämpfl Florian, Kanawade Rajesh, Riemann Max, Knipfer Christian, Stelzle Florian, Schmidt Michael

**Affiliations:** Institute of Photonic Technologies, Friedrich-Alexander-Universität Erlangen-Nürnberg, Konrad-Zuse-Str. 3/5, 91052 Erlangen, Germany; Erlangen Graduate School in Advanced Optical Technologies, Friedrich-Alexander-Universität Erlangen-Nürnberg, Paul-Gordan-Str. 6, 91052 Erlangen, Germany; Department of Oral and Maxillofacial Surgery, Friedrich-Alexander-Universität Erlangen-Nürnberg, Glück Str. 11, 91054 Erlangen, Germany

**Keywords:** Diffuse reflection, Angiographic imaging, Cutaneous tissue phantom

## Abstract

**Background:**

Diffuse reflection imaging could potentially be used to recover the superficial microvasculature under cutaneous tissue and the associated blood oxygenation status with a modified imaging resolution. The aim of this work is to deliver a new approach of local off-axis scanning diffuse reflection imaging, with the revisit of the modified Beer–Lambert Law (MBLL).

**Methods:**

To validate this, the system is used to recover the micron-scale subsurface vessel structure interiorly embedded in a skin equivalent tissue phantom. This vessel structure is perfused with oxygenated meta-hemoglobin solution.

**Results:**

Our preliminary results confirm that the thin vessel structure can be mapped into a 2-D planar image. The distributions of oxygenated hemoglobin concentration ($Ct_{HbO_{2}}$) and deoxygenated hemoglobin concentration ($Ct_{RHb}$) can be co-registerated through the MBLL upon the CW spectroscopy, the scattering issue is addressed in the reformed MBLL. The recovered pattern matches to the estimation from the simultaneous optical coherence tomography studies.

**Conclusions:**

With further modification, this system may serve as the first prototype to investigate the superficial microvasculature in the expotential skin cancer loci, or a micro-lesion of vascular dermatosis.

## Background

Metabolism in the lesion of cancer, or vascular dermatosis is enhanced and therefore, this region has to be supplied by more blood vessels to deliver oxygen. Monitoring the microvasculature density in areas with increased angiogenic activity is one of the most convincing criteria to determine the growth of a tumor in its initial/recurring stage, or the deterioration of the angioma and vascular dermatosis in some cases [1, 2]. Non-invasive means of vessel imaging mainly adopt the radiological and optical technics such as computer assisted tomography (CT), functional magnetic resonance imaging (fMRI), and optical coherence tomography (OCT) [3, 4]. Using these technologies, the number of biopsies could be reduced by providing a first impression guidance. These approaches have already been examined on in-vivo studies to facilitate the monitoring of the blood flow/metabolism in expotential lesion of breast cancer, traumatic brain injury, and heart defect [5–7]. Focusing on their uses on the angiographic imaging of the cutaneous tissue, a speckle-variance OCT (sv-OCT) could effectively map the pattern of microvasculature/micro-circulation in the epidermis with a depth-resolution and assist the diagnosis of skin cancer [8].

However, these modalities can hardly illustrate the blood oxygenation, while it is an extraordinarily meanful optical biomarker to distinguish a tumor progression from e.g. the normal inflammation [2]. Recent researches on OCT technics did not only involve a task to feasibilize monitoring the oxygenation via an ultra-high-resolution OCT and advanced means of pattern recognition [9], but also to evaluate the hemoglobin concentration (change) via a spectroscopic OCT [10, 11]. But none of these results could be acquired without an intensive signal processing or sophiscated system alignment. Consequencely, a disproportional investment on devices might obstacle their dissemination from labs into clinics.

Diffuse optical technics have evolved as a powerful tool to functionally substitute the expensive modalities in some specific diagnostic scenarios. It adopts the diffuse remitted light out of the tissue, which is informative about the blood oxygenation (changes). Such information is oftenly related to the chromspheres in the vasculature or extracellular matrix [12]. In principle, diffuse optical technics allow for recovering specific chromospheres in tissue, primarily the oxy- and deoxygenated hemoglobin. Especially the diffuse optical imaging (DOI) could have substituted the approaches, like fMRI and CT, in the lesion analysis of breast cancer [13, 14] traumatic brain injury [15] and heart defect [16]. Technically, the diffuse optics has been transited from employing single-point probe over source-detector pairs toward planar sensing array [12]. Research has also been conducted onto different derivations of diffuse optics, like diffuse optical tomography (DOT) [14], time gated scanning diffuse optical imaging method [17] etc. Compared to other modalities, diffuse optical technologies provide an enhanced sensitivity to absorbing targets in depth concurrently with an achievable temporal resolution and cost efficiency. However, despite the impressive development in the technics, insufficiencies still exist, including inadequate spatial resolution, challenges in artifacts removal, and complicated inverse reconstruction [12, 18].

Our ultimate interest is to develop a diffuse reflection approach to provide an angiographic imaging of the cutaneous microvascular pattern, or compromisingly the micro-lesion (size ≈100 μm) under skin. Relevantly for similar purpose, a novel modification of Laminar Optical Tomography (LOT) could have given a high resolution 3-D reconstruction of such a subsurface target, namely the vascular network. However, the confocal-type design of LOT is costly. Even under this cost, it is only capable to recover a subsurface target in size of 100–200 μm [19]. Moreover, the use of CW spectroscopy in diffuse optical study is deliberately avoided, for reason that the prediction of the light propagation in depth of the turbidding tissue is difficult. Because of this, frequency domain spectroscopy is more widely studied, even its algorithm might increase the cost of hardware and computational power. In our study, the target to be recovered is located superficially. This means, the path length of light is limited to the subsurface and could be characterized by using CW spectroscopy. Mathematically, the path length of light through the turbidding media could be yield through the modified Beer–Lambert Law (MBLL), if the scattering issue of the target is restricted.

The objective of this work focuses on improving the imaging resolution of the diffuse optics to micron-scale, and meanwhile with a simple strategy of signal processing and system alignment. In the previous study as reported elsewhere [20], we have introduced the prototype system. We also provided a proof of conception of adapting the MBLL to the local-off-axis alignment. As the result, the micron-scale target can be projected into a 2-D planar image with high pixel density. In this work, we shall more specifically investigate how the imaging resolution could be correlated to the induced change in the scanning scheme. Again to validate this, it is first used to recover a micron-scale superficial vessel structure interiorly embedded inside a skin equivalent phantom. Further more, we would give a modification of the MBLL algorithm to better address the impact from the scattering issue of the phantom.

**Fig. 1:**
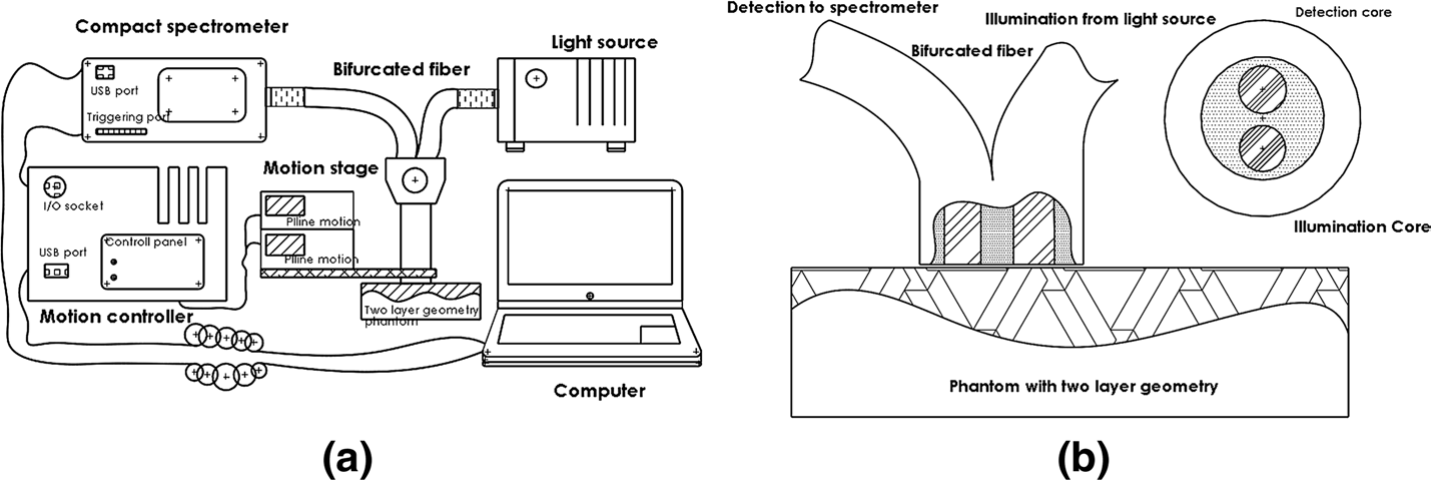
**a** Schematic of the local off-axis scanning diffuse reflection imaging system: the bifurcated optical fibre as off-axis module; the motorized stages as scanning module; and a spectrometer as detection module. It consists of (1) motion stages, (2) bifurcated optical fibre, (3) halogen light source, (4) compact spectrometer, (5) controller, (6) triggering cabel, and the other integrated control unit. **b** Schematic of the bifurcated optical fibres. The apreture separation defines a geometric off-axis between the illumination and the detection cores

## Methods

### Experimental setup of sDRI

The local off-axis scanning diffuse reflection imaging system is schematically shown in Fig. 1a. It consists of three modules: optical off-axis module, scanning module and detection module. Components of these three modules are electromechanically coupled together for a robotic 2-D planar scanning procedure. The optical off-axis module attributes to a customized bifurcated optical fibre (Ocean optics Co., USA). This fibre ensembles an illumination core and a detection core with a core diameter of 50 μm and a aperture separation of 125 μm from central to central, with all arms terminated with SMA 905 connectors. Steadily on each measuring point, light is coupled from a 20 W halogen light source (HL-2000, Ocean optics Co., USA) through the illumination core into the object sample, as it is then diffusely remitted into the detection core toward a scientific grade spectrometer (QE 65000, Ocean optics Co., USA), as shown in Fig. 1b. For the simultaneous scanning of the ensemble end of the optical fibre over a X–Y plane upon the surface of the object sample, the optical fibre is adapted on the motorized stages (M-663 ®motion stage, Physik Instrumente GmbH, Germany). This stage has a travel range of 18 mm, 2 μm minimal motion step length and provides a precise motion capacity with a close loop motion cycle to take the inventory of the motion accuracy. Two orthogonally aligned motion stages enable a combined scanning along x and y direction. The spectrometer and the motion stages are synchronously triggered with a controller (C-867.260 PILine ®Controller, Physik Instrumente GmbH, Germany) through the digital I/O lines (TTL signal, analog: 0–5 V) to start/stop action. On each measuring point, one acquisition is gated by a triggering signal after the stage braked. In the prototype system, the entire cycling of motion, brake, trigger, acquisition and delay on each single measure point lasts 30 ms.

**Fig. 2:**
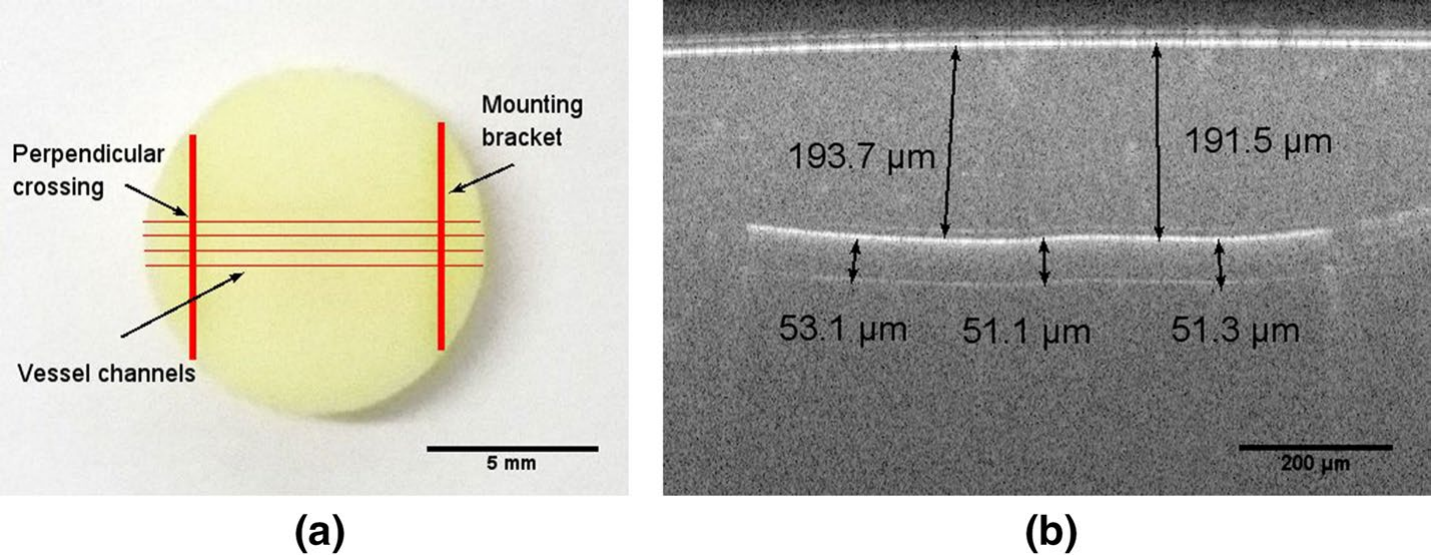
**a** Picture of skin equivalent tissue phantom with four separate rows of vessel channels indicated and **b** OCT B-scan image of cross section passing along the vessel channel in tissue phantom and the overlaid measured values of the geometry

### Skin equivalent phantom model

The preparation of a single layer skin equivalent tissue phantom is reported elsewhere [21]. Principally, we embed copper wires in the matrix material, and eliminate them by using ferric chloride. The matrix of the phantom is constructed out of polyurethane, titanium oxide and India ink [22]. The matrix material allows for a reduced scattering and absorption coefficients of $\mu _{s}^{\prime }\,=\, 3.1\,\, {\rm cm}^{-1}$ and $\mu _{a}\,=\, 0.13\,\, {\rm cm}^{-1}$ at the wavelength of 660 nm, which replicate the representatives values from the Caucasian skin [22]. These optical parameters are not alteratedduring the fabrication procedure [21]. To simulate the structure of the superficial microvasculature under skin, we embed four separate rows of interior channels with an aimed diameter of 50 μm and at an average depth of about 200 μm to the surface (the finished phantom is shown in Fig. 2 [21]). Hillman has reported elsewhere [19] a similar phantom, where a human hair was embedded as the targeted absorber. In our work, fresh oxygenated meta-hemoglobin solution (hemoglobin human, Sigma-Aldrich. Co, USA) with a concentration of 120 g/L is included into the hollow vessel channels as the light absorber. These simulate the micro-circulation in cutaneous vasculature.

**Fig. 3:**
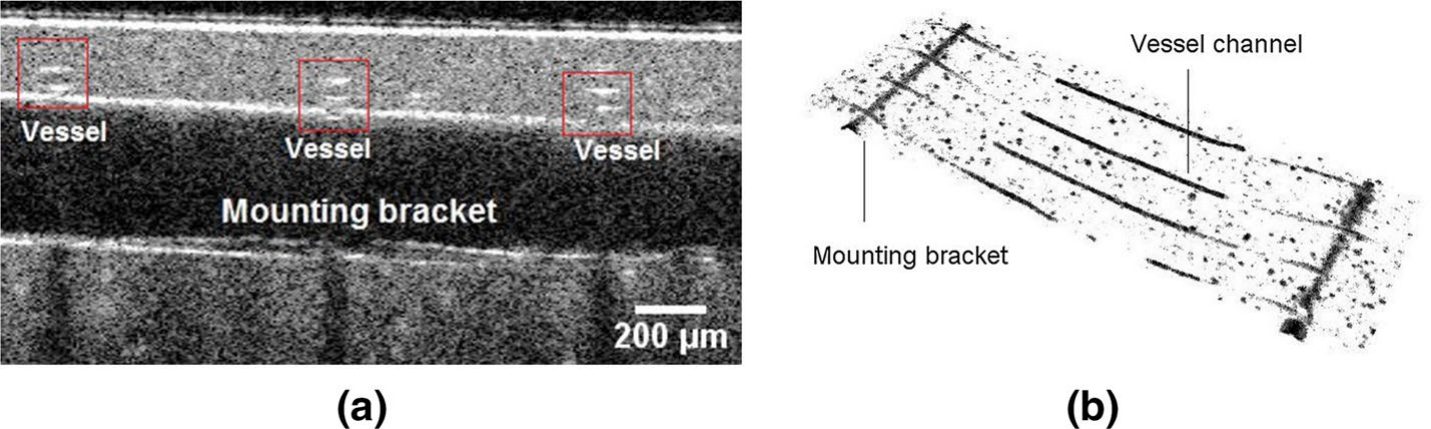
**a** OCT B-scan image of the cross section passing along a mounting bracket across the the vessel channels, the cross section of the vessel channels are* circled* by* red rectangular*. **b** OCT volume view of the segmentated vessel structures. They include four separate rows of vessel channels perpendicularly crossing over two rows of mounting brackets

### Scanning scheme and paradigm

Experimentally, the geometry of the generated vessel structures is independently co-checked by using a SD-OCT ( center wavelength 1300 nm, objective: LSM, Medium-Sensitivity Mode: 48 kHz; Telesto-II-SP1, Thorlabs GmbH, Germany). Read from the OCT B-scan image (see Fig. 3a) and 3-D volume view (see Fig. 3b) [21], the generated vessels perforate through the phantom slab, and perpendicularly cross into the mounting bracket, thus forms a physiological complexities of perpendicular crossing. Likewise, we scan over the area of interest with highest structural complexity, namely the perpendicular crossing, via sDRI, and compare to the OCT image. An orthogonal crisscross liked pattern is assumed to be recovered to indicates this perpendicular crossing. Besides, to investigate the influences from the motion step length of the scanning scheme on the imaging resolution, we scan over a certain region with an induced change of motion step length from 5 over 10–50 μm.

### Data analysis and image recovering

**Fig. 4:**
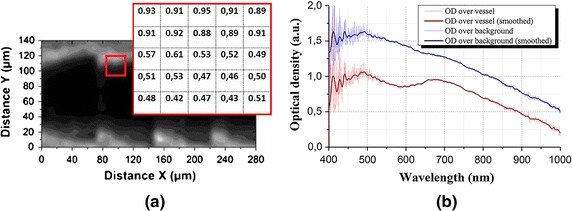
**a** A 2-D planar imaging of optical density arranged to their corresponding position in the coordinate of scanning scheme. **b** An OD-wavelength spectra showing the contrast of of the optical density between over around the scattering matrix (*blue curve*) and vessel included with hemoglobin solution (*red curve*), the *red curve* shows a lower value of optical density with the wavelength dependency, as the hemoglobin absorbs more light

The detection core of the optical fibre is manipulated to a scientific grade compact spectrometer to register the intensity-wavelength spectra. The values of intensity *I* from these spectra are abstracted numerically at the wavelength of 660 nm. We convert them into the key values of optical density *OD* by normalizing to the reference intensity $I _{0}$ (see Eq. 1), and correspond them into their position in the scanning scheme. Thus, an image of gray scale is plotted by iteratively matching the values of optical density onto a 2D planar coordinate (see Fig. 4a).

1 $$\begin{aligned} OD^{\lambda _{1}} = \ln \left( \frac{I_{0}}{I}\right) , OD^{\lambda _{2}} = \ln \left( \frac{I_{0}}{I}\right) \end{aligned}$$

Figure 4b shows the optical density spectra of over around the scattering matrix material and the absorbing vessel structure respectively. The contrast of the gray scale image stems directly from the distance between these two characterizing spectra of optical density. With this, the recovered image can differentiate the absorbing target against the scattering background.

The values of optical density are further translated into the oxy-hemoglobin concentration $$Ct_{HbO_{2}}$$ and the deoxy-hemoglobin concentration $$Ct_{RHb}$$$$\left[\frac{mol}{l}\right]$$ by solving the modified Beer–Lambert Law (MBLL). The $$\varepsilon ^{\lambda _{n}}_{HbO_{2}}$$ and $$\varepsilon ^{\lambda _{n}}_{RHb}$$$$\left[\frac{l}{cm.mol}\right]$$ represent respectively for the molar extinction coefficient of oxy- and deoxy-hemoglobin at specific wavelengths. To calculate the oxy- and deoxyhemoglobin concentration, data processing must be performed at two or more different wavelengths around the isosbetic point. In this case, we choose the wavelengths of 660 and 940 nm, as they are commonly used in pulse oximeter.

$$\begin{aligned} OD^{\lambda _{i}} = \left[ \varepsilon ^{\lambda _{i}}_{HbO_{2}} *Ct_{HbO_{2}} + \varepsilon ^{\lambda _{i}}_{RHb} *Ct_{RHb} \right] *L \;*f _{DPF}^{\lambda _{i}} + G _{optode}. \end{aligned}$$

The $L *f _{DPF}^{\lambda _{n}}$ in the equation stands for the differential path length of light inside the tissue. This path length is mostly determined by the scattering issues of a turbidding material as well as the spatial separation between illumination source and detector. The translation through MBLL also undergoes an approximation of the geometric path length from illumination to detection. In this study, we simplify this path length as source-detector distance, which means, the geometry factor $G _{optode}$ is neglected, and the values of $Ct_{HbO_{2}}$ and $Ct_{RHb}$ on the calibration scale bar are not yet calibrated to their exact values.

## Results

### Planar image of optical density

**Fig. 5:**
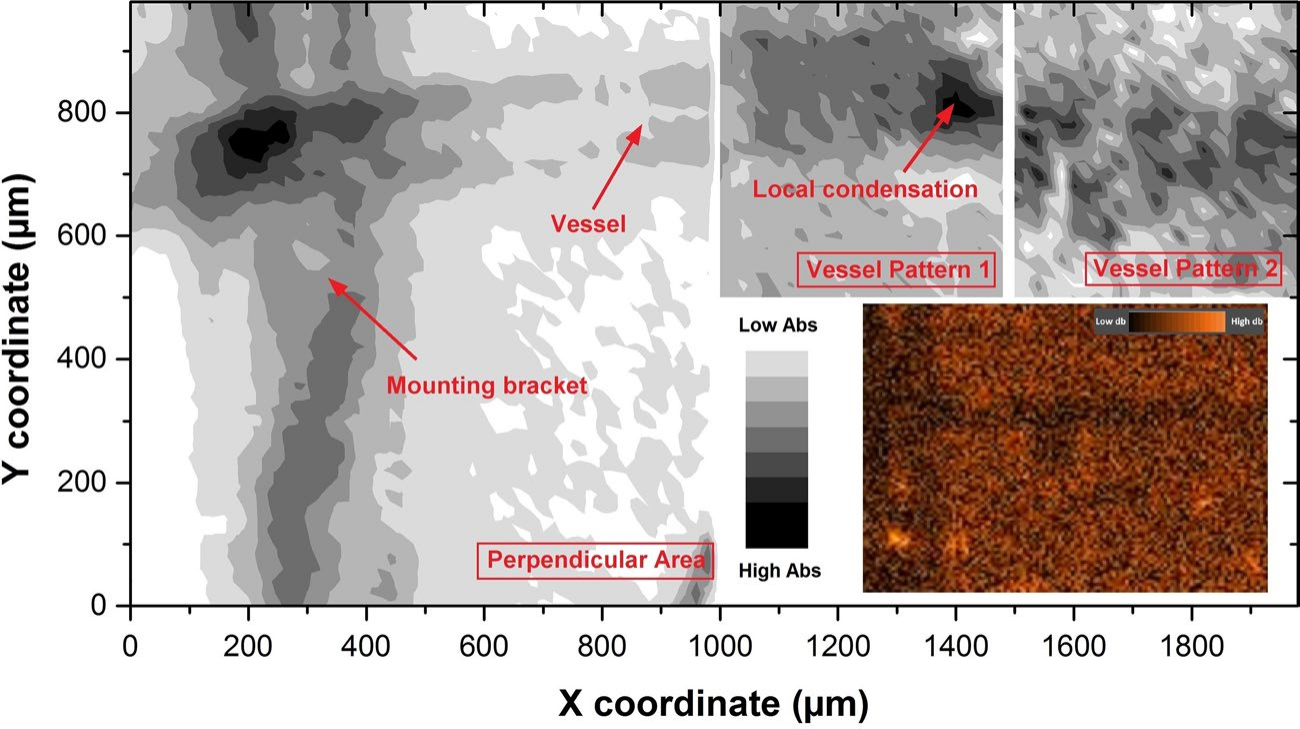
Recovered image of vessel pattern around the area of interest with a motion step length of 20 μm. The boundary highlights the possible interface from the absorbing vessel channel to the scattering background of matrix material; an estimated OCT image is shown at* right bottom*

We scan over around the region of perpendicular crossing with a motion step length of 20 μm. This motion step length gives the best balance between the resolution (as stated later) and the time duration for 1 scan. As the result of this procedure, we receive an image with a field of view (FOV) of 1 × 2 mm. Figure 5 illustrates this planar top view over the area of interest. The obtained pattern is stitched out of three regions: *perpendicular area*, *vessel pattern 1* and *vessel pattern 2* along one vessel channel (as labelled from left to right in Fig. 5). The region *perpendicular area* contains 100 × 100 pixels (from 100 × 100 measure points in scanning scheme), the region *vessel pattern 1* and *vessel pattern 2* contains 50 × 50 pixels. For each pixel, the length and the width equal to the motion step length in the scanning scheme (20 μm). The gray scale grades the absorbance from high level in dark to low level in white, with no spatial filtering or smoothing applied.

First, we discuss on this general top view, the gray shaded zone demonstrates that the optical fibre scans over an absorbing target, mainly the vessel channel and the mounting bracket. Read from the image, we recognize a vessel channel horizontally perforating through the three segmentation regions with a contrast against the scattering background. The geometry of the perpendicular crossing is patterned in region *perpendicular area* on the left, where we find the vessel in horizontal and the mounting bracket in vertical direction. These two patterns cross perpendicularly into each other, thus form a crisscross liked pattern, as we assumed. Figure 5 also shows an estimated image recovered with OCT at right bottom. In the OCT image, brighter pixels indicate the target with higher reflectivity, while the darker pixels for lower reflectivity. This image show the same perpendicular cross, whose morphology matches to that in the sDRI image.

### sDRI versus SD-OCT image

**Fig. 6:**
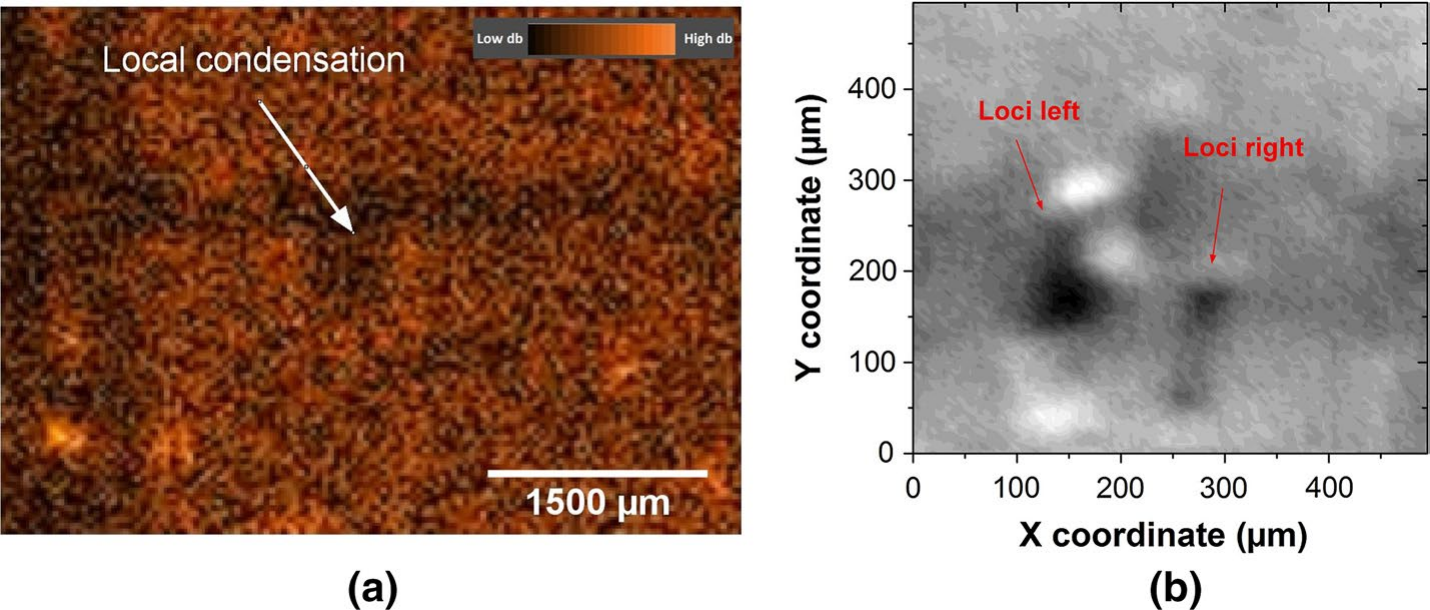
**a** OCT topview of a 50 μm diameter vessel channel with a labeled fleckle induced by condensation of hemoglobin solution and **b** the vessel channel recovered by sDRI around the same area of local condensation with two labeled spots representing the condensation of hemoglobin

Notably, there exists a dark spot in the middle of the OCT image (see Fig. 6a). This shadow could be caused by the local condensation of the included hemoglobin solution in vessel, which absorbs more portion of light. Principally, hemoglobin solution flows flows into the air bubbles in phantom matrix (micron-scale), which are opened to the vessels. These air bubbles, where the condensation occurs, are mistakenly introduced. However, this could be a good target to test, whether the sDRI could capture the small target. As a matter of fact, this local condensation could have mimicked the structure of e.g. tumor nodules in skin. The sDRI image (see Fig. 6b) enlarges this dark spot on the same position, that consistents to the shadow in OCT image. In both sDRI and OCT image, this fleckle is located at about 1200 μm away from the main body of the mounting bracket. In the sDRI image, the pattern of the local condensation is even more detailed splitted into two corresponding loci on the left and on the right (as labelled in Fig. 6b).

### Impact from the scanning scheme

**Fig. 7:**
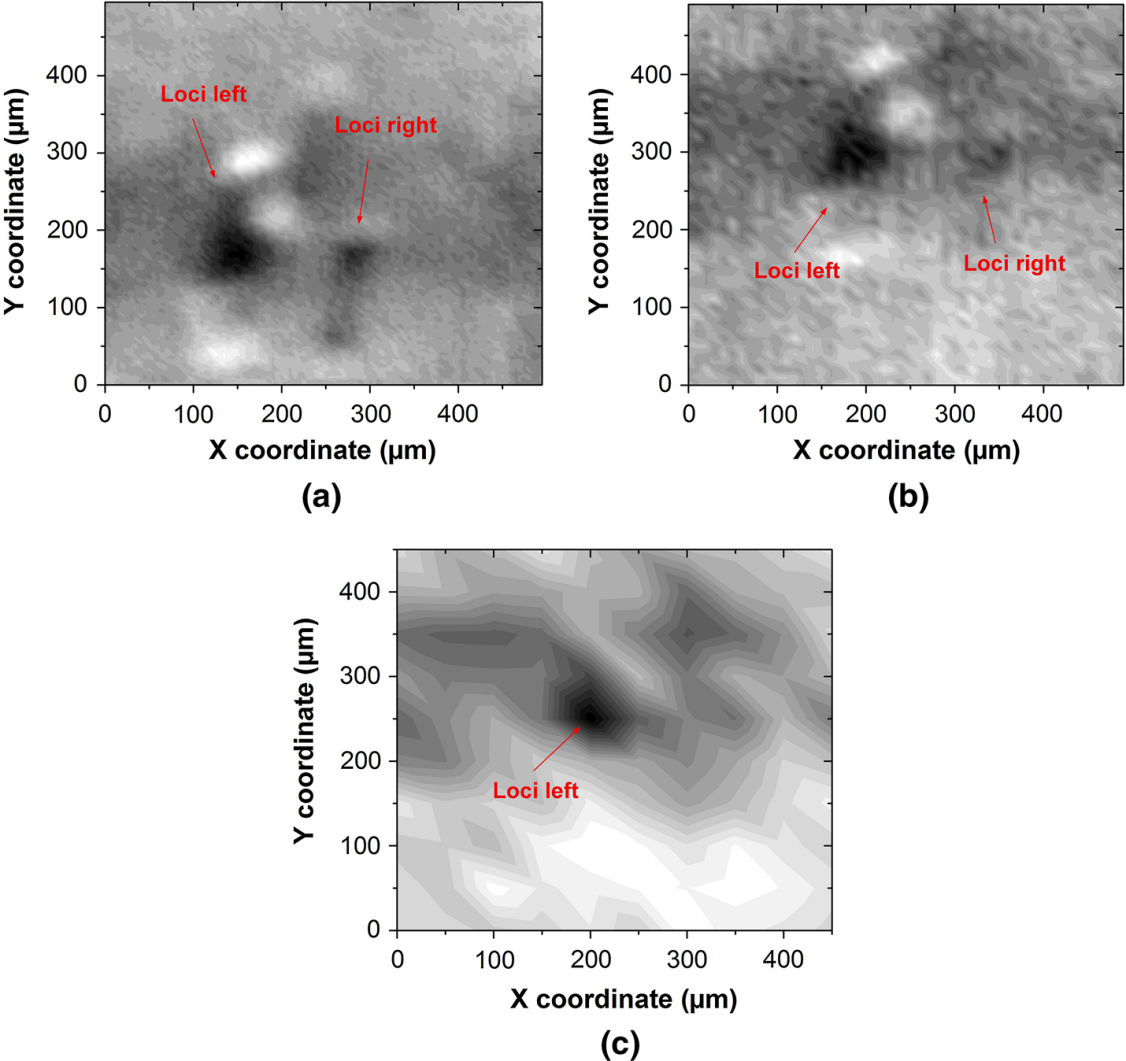
Recovered image of the 50 μm diameter vessel channel measured with an induced change of the scanning scheme of** a** 5 μm step length,** b** 10 μm step length and** c** 50 μm step length

The amount of the measuring points in scanning scheme decides the pixel density in the recovered image. We further discuss the impact from the scanning scheme on the recovering capacity. Figure 7 demonstrate the recovered images of the local condensation peripherally with a changing of the motion step length of the scanner. The location of the local condensation is regarded as the mark to assess, whether the sDRI has the capability to capture the small target. Therefore, both spots of the local condensation are deliberately observed to rate the imaging resolution. The image recovered with a motion step length of 5 μm is shown in Fig. 7a. The boundary of targeted vessel channels and spots of the local condensation are properly recovered with a clear contrast against the scattering background. The shadow zone, which represents the vessel channel, ranges from 200 to 300 μm on Y coordinate. Inside this shadow, it is showing more elevated values of optical density against the background.

We increase the motion step length to 10 μm and scan over around the same region. Compared with the image recovered with a step length of 5 μm, this image (see Fig. 7b) reveals more blur. It loses, or even shifts a detailed reconstruction of the boundary between the absorbing and scattering area. The spots of the local condensation preserve only partially, as the loci on the right is almost vanishing from the image (as labeled in Fig. 7b). In both Fig. 7a, b, the optical density decreases from a peak value of 0.25 a.u. upon the absorbance maximun to the absorbance minimun of around 0.1 a.u., with a drop of 60 %. Therefore, the gray scale differentiation of the image is turned out to be high. Contrastly in the Fig. 7c, the blur is totally becoming a mosaic under a larger motion step length of 50 μm. Under this motion step length, the scanning system could have even lost its capacity of resolving both two spots of the local condensation.

**Fig. 8:**
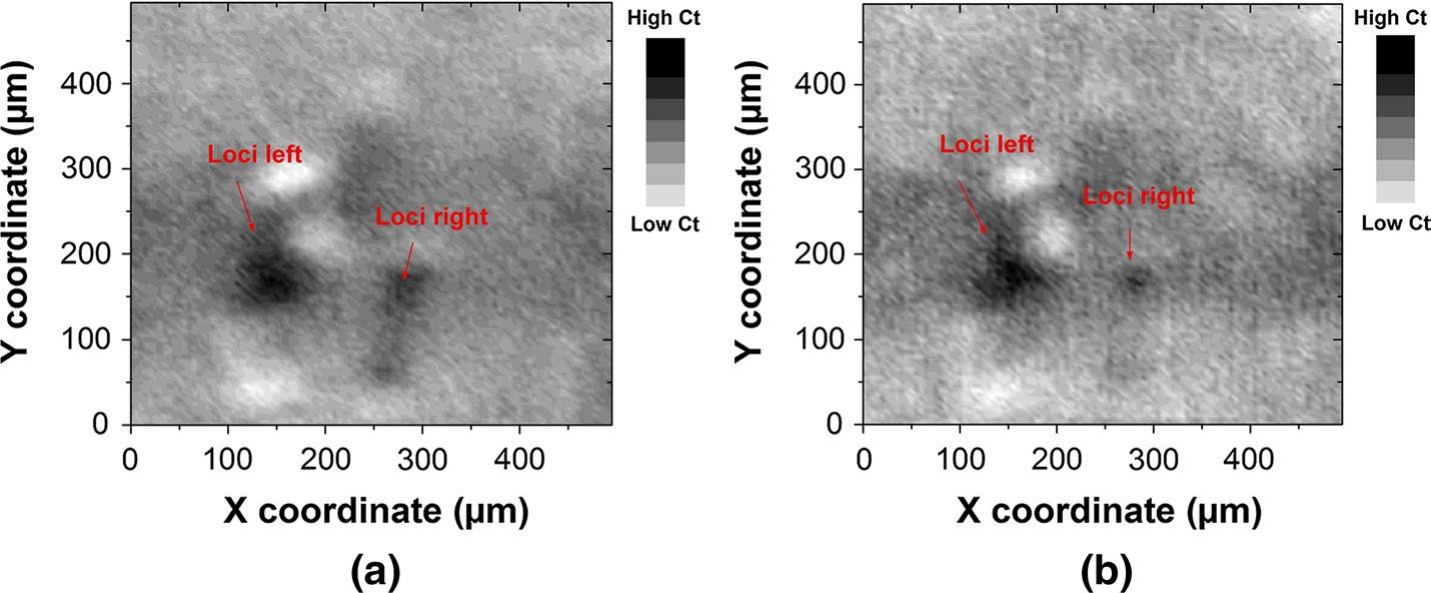
Recovered image of vessel channel demonstrated as the distribution of **a** $Ct_{HbO_{2}}$ and **b** $Ct_{RHb}$

### Planar distribution of $Ct_{RHb}$ and $Ct_{HbO_{2}}$

After our initial tests on recovering the microvascular pattern, we attempt to recover the distribution of the hemoglobin concentration. The measured values of optical density are converted into the absolute values of $Ct_{HbO_{2}}$ and $Ct_{RHb}$ through the modified Beer–Lambert Law. Figure 8 shows the recovered image of $Ct_{CHbO_{2}}$ and $Ct_{RHb}$ distributions with a motion step length of 5 μm. Image of $Ct_{CHbO_{2}}$ (see Fig. 8a) indicates very slight changes compared with the image of optical density distribution in (Fig. 7a). The recovering of the local condensation survives after the translation and dovetailes. On the other side, both spots of local condensation fade in the image of $Ct_{RHb}$ (see Fig. 8b) compared with those in image of $Ct_{HbO_{2}}.$

As the native function of diffuse optics, the sDRI system implements a reconstruction of $HbO_2$ and *RHb* concentration distributions. Based on the fact, that the injected hemoglobin in the phantom is mostly oxygenated with its exposure to air, the spots of the local condensation in $Ct_{RHb}$ image is expected to be alterated compared to that in the image of $Ct_{HbO_{2}}.$ This inference is warranted by the facts by comparing Fig. 8a, b. It means, sDRI system can distinguish the oxygenation status of the included hemoglobin, but only qualitively since the path length factor has not yet been calibrated. Nevertheless, the sDRI system provides more information about tissue oximetry, than it can be gained from OCT without spectroscopic alignment, or functionally less processing intensive compared with a spectroscopic OCT, like described in Ref. [11].

## Discussion

The recovered sDRI image maps the defined vessel channels against the scattering background and reconstructs the geometric complexity of perpendicular crossing in tissue phantom. By comparing to a simultaneous study using a SD-OCT, it is proved that the system can resolve a micron-scale target, i.e. the pattern of the local condensation, under a fine incrementation of the scanning scheme. This dimensional scale has not yet been discovered by other diffuse optical approaches, according to our knowledge [13–15]. Compared to our previous study as reported elsewhere [20], we have proved that the motion step length is an influential aspect. The imaging resolution is inversely proportional to the motion step length of the scanning scheme. With such a simplified setup, it could achieve an equivalent resolution, as that of the LOT [19]. The core competence is not the hardware, instead, it is stated as the recall back of the modified Beer–lamber Law upon the local off-axis aperture separation. Within the MBLL based algorithm, it is becoming practicle to recover the light attenuation in the superficial target by using a CW spectrometer. CW spectrometer is cheaper and requires less expensive assisoires, like laser/coherrent light source. This means, it would help to avoid a disporportional investiment, while the other skin angiographic imaging apporaches, such as OCT or LOT, can’t.

Furthermore, the most established diffuse optical approaches measure the values of the oximetric parameters $\delta Ct_{HbO_{2}}$ and $\delta Ct_{RHb}$ instead of their absolute value, since the factor of opotode geometry and the scattering issue can not be neglected. As the result, it is almost meanless to calibrate the differential path lenght factor when the distance between opotodes exceeds about 2.5 cm, quoted from G. Strangman’s clearance in his review work [18]. A local off-axis of the two ensembled cores with a separation of 125 μm is subversive to the common boundary condition of MBLL. Calibrating the path length factor, or assigning an absolute value of hemoglobin concentration through MBLL is theoretically possible, and therefore, becomes a purposeful topic for future works.

However, this method does not come without limitations. Firstly, vessels are dimensionally overestimated than the actual defined values of 50 μm in diameter. The width of the gray shaded vessel pattern is over around 100 μm on Y axis, almost double size of the actual width. This problem is commonly found in other diffuse optical approaches [13, 18]. To address this challenge, images should be de-convoluted individually according to their calibration criteria of optical properties, e.g. absorption coefficient $\mu _{a}$ and reduced scattering coefficient $\mu _{s}^{\prime}.$

Different from our previous work, we reformed the scattering issue $F_{scatt}$ in the extended form of MBLL (see Eq. 2) in this work. The general equation of the MBLL is shown as the following.

2 $$\begin{aligned} OD^{\lambda _{i}} = \left[ \varepsilon ^{\lambda _{i}}_{HbO_{2}} *C_{HbO_{2}} *f _{DPF HbO2}^{\lambda _{i}} + \varepsilon ^{\lambda _{i}}_{Hb} *C_{Hb} *L *f _{DPF RHb}^{\lambda _{i}} \right] + G _{optode} + F_{scatt} \end{aligned}$$

where the differential path length factor DPF in the scattering issue $F_{scatt}$ could be characterized through the variation of the diffusion equation (see Eq. 3) In the previous work [20], we have simply excluded the $F_{scatt},$ as it was writen as a constant and integrated into the geometry factor $G_{optode}.$ To distinguish from the previous work, we re-programm the MBLL by replacing the constant value of $F_{scatt}$ by the equation as shown in the following. The parameters of absorption coefficient $\mu _{a}$ and reduced scattering coefficient $\mu _{s}^{\prime}$ are read from the spectrum of, in this case, the phantom slab. The reconstructed image does not show significant morphological difference by comparing the figure to those in our previous publication [20], as the $F_{scatt}$ remains constant for the single equation. However, each pixel includes a better calibrated value of hemoglobin concentration after the reformation. The $F_{scatt}$ shall no longer be a simple unchanged value. Instead, the $F_{scatt}$ shall be then correlated to the skin type (their optical parameters). With its variation under different skin types, the MBLL could give us the exact absolute values of the oxymetric parameters of the hemoglobin concentration. It would be the orientation of our work in the upcoming period, to fix the $F_{scatt}$ within the change of skin (phantom) types.

3 $$\begin{aligned} DPF^{\lambda _{i}} = \frac{1}{2}\left(\frac{3\mu _{s}}{\mu _{a}}\right)^{1/2} *\left[1-\frac{1}{(1+d(3\mu _{a}\mu _{s}^{1/2}))}\right] \end{aligned}$$

Experimentally, the work shall be conducted onto evaluating the dimension of vessel channel experimentally on tissue phantoms, with optical properties that cover the range from those of caucasian to the negroid type. In details, the $F_{scatt}$ shall be calibrated with the change of the optical parameters of the phantoms, which simulate caucasian to the negroid skin cutis (epidermal and dermal). Computational simulations (Monte Carlo inversion) of light propagation in turbid media shall be proceeded to verify the differential path length of light in tissue/digital phantom.

The constructed sDRI system is yet still the first prototype. The primary problem is that the scanning procedure must be accelerated, so as to provide an efficient FOV within a shorter duration. As described in the report, a delay of at least 30 ms is needed upon each measuring point in the scanning scheme. During this 30 ms, despite the dead/rise time of the compact spectrometer, the most duration is spent on reading the GSC command (the machine language that drives the motion stage) from the software panel onto the motion controller. The GSC language is written as the function in Dynamic-Link library (DLL), which could be directly extracted by using MATLAB. By doing this, the delay for reading command could be evitable. In the latest test on sDRI system, we have succeeded shortened this delay to less than 5 ms. In the future work, we shall attempt to modify the sDRI by using a compact NIR spectrometer module (CCS175/M NIR compact spectrometer, Thorlab, Germany) with shorter dead/rise , or integration time. This helps to further accelerate the scanning procedure.

The skin equivalent phantom is unique. This phantom features the scattering nature of skin cutis and meanwhile the vascular geometry. This helps to provide a better insight into the imaging capability of recovering the oximetry parameters and resolution. In one sense, we do not only present the diffuse reflection imaging approach, but also introduce a novel methodology to perform the phantom validation. However the phantom can not simulate a real cutaneous tissue on all aspects. For instance, the fingerprints can affect the reflectance of light. Unfortunately, to fabricate such a surface topography to mimic the fingerprint is challenging. Besides, the defined structure of the superficial microvasculature in the phantom is comparatively simple with a homogeneous inclusion and matrix material. Ex-vivo and in-vivo studies require more considerations about the heterogeneity of other physiological structures and chromospheres in skin. For example, the epidermis of a real human tissue contains melanin, which has a constant extinction coefficient that affects on the reflectance of skin. Those disruption could be reduced through a data acquisition within a proper wavelength selection. Furthermore, overlay of the vascular network under tissue is not uncommon, but can not be modelled with the previous phantom of ours. Existing multilayer/micro-fluidic chip based phantom with embossed vascular structure could have simulated this nature. But most of them include an adhesive sealing material between layers, that disrupt the optical properties [23]. A crucial topic for the future works will be to fabricate a self-packaging phantom with multiple layers to mimic the cutaneous cutis and vascular network.

## Conclusion

We have presented the instrumental setup of our new approach of sDRI and the results on recovering the superficial vascular pattern through the phantom validation. The scanning scheme with a fine motion step enabled a reconstruction of the vessel channels $(\phi = 50\,\,\upmu \mathrm{m})$ and a geometric complexity. It is to be noticed that the reconstructed pattern is slightly overestimated than the exact size of target. Nevertheless, the results proved a modified imaging resolution correlated to a fine motion step length of the scanning scheme. Our system could be regarded as a promising prototype to improve the imaging resolution of the diffuse optical technics.

With further modification, our system could prospectively be used to image the microvasculature and the associated oximetry in skin. Or compromisingly, it can already recover a micron-scale absorbing superficial target under turbidding media, such as a micro-lesion of the expotential skin cancer. Unlike the formal research on diffuse optics, we recall back the use of the modified Beer-Lambert Law and the application of the CW spectroscopy. The oximetry parameters of $Ct_{HbO_{2}}$ and $Ct_{RHb}$ is mapped into a 2-D planar image irregardless of the uncalibrated path length factor. Anyway, the local off-axis of the illumination-detection pair subverses to the boundary condition of the modified Beer-Lambert Law directly, and enables an assignment of the absolute value of $Ct_{HbO_{2}}$ and $Ct_{RHb}.$ The scattering issue could be addressed by the general equation for differential pathlength factor.

The future works include a series of phantom studies to calibrate the differential path length factor and its variance with the optical properties of the turbidding material. To adapt an inverse reconstruction algorithm to the local off-axis setup for depth discrimination is planned. Last but not least, to construct a micro-fluidic based multilayer phantom with overlaying microvascular network is a part of the future work as well.
